# Supplementary material for: Vascular imbalance in polycystic ovary syndrome: Insights into endothelial dysfunction
Source: PLoS One. 2025 Sep 5;20(9):e0332030. doi: 10.1371/journal.pone.0332030 (PMC12412924; doi:10.1371/journal.pone.0332030)

SAMPLING

The sample size of this study was determined by power analysis. According to the calculation made using the G*power 3.1 program; the sample size was determined to be at least 84 (42 in each group) with an effect size of 0.73, margin of error of 0.05, confidence level of 0.95, and representative power of the universe of 0.95 (Faul et al., 2009).

Faul F, Erdfelder E, Buchner A, Lang AG. Statistical power analyses using G*Power 3.1: tests for correlation and regression analyses. Behav Res Methods. 2009;41: 1149-1160. doi: 10.3758/BRM.41.4.1149.

APPENDIX - G.Power Program Output

t tests - Means: Difference between two independent means (two groups)

**Analysis:**	A priori: Compute required sample size		
**Input:**	Tail(s)	=	One
	Effect size d	=	0.73
	α err prob	=	0.05
	Power (1-β err prob)	=	0.95
	Allocation ratio N2/N1	=	1
**Output:**	Noncentrality parameter δ	=	3.3452803
	Critical t	=	1.6636492
	Df	=	82
	Sample size group 1	=	42
	Sample size group 2	=	42
	Total sample size	=	84
	Actual power	=	0.9528002

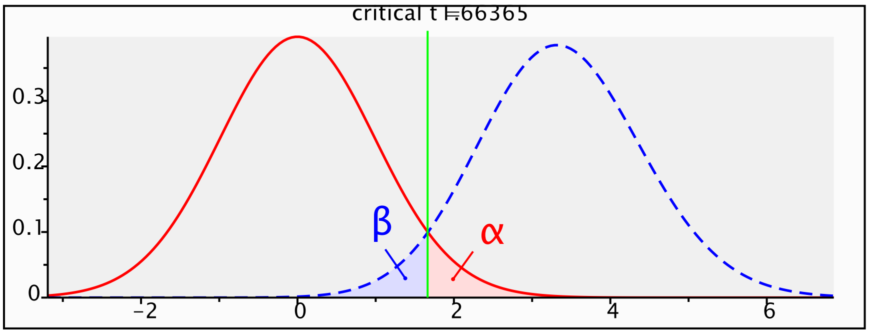

Supplement: S1 File — (PDF) [file pone.0332030.s001.pdf]
