# Supplementary material for: Vascular imbalance in polycystic ovary syndrome: Insights into endothelial dysfunction
Source: PLoS One. 2025 Sep 5;20(9):e0332030. doi: 10.1371/journal.pone.0332030 (PMC12412924; doi:10.1371/journal.pone.0332030)
Supplement: Attachment — Submitted filename: Response_to_Reviewers_auresp_2.docx [file pone.0332030.s005.docx]

**RESPONSE TO REVIEWERS**

**Response to Reviewer 3**

***### Reviewer 3 Comment 1: Clearly state the novelty of your study in the Introduction.***

We thank the reviewer for their valuable comment. As suggested, we would like to highlight that the novelty of our study has been articulated in the Introduction section:

“Although the physiological roles of NO, apelin, noradrenaline, and prostacyclin have been individually investigated, no study to date has systematically examined their combined contribution to vascular tone imbalance in PCOS. Furthermore, their collective diagnostic value in distinguishing PCOS patients from healthy individuals remains largely unexplored. This study addresses this gap by providing an integrative evaluation of these markers, thereby offering a more comprehensive approach to understanding endothelial dysfunction in PCOS.”

However, if the current phrasing does not sufficiently convey this point, we would be pleased to revise the text or include an additional sentence to ensure the novelty is clearly and explicitly stated.

***### Reviewer 3 Comment 2: Briefly justify the rationale for selecting the four specific biomarkers.***

We thank the reviewer for this valuable suggestion. To address this comment, we have added a sentence to the Introduction section clarifying the rationale for selecting the four specific biomarkers:

"These four biomarkers were selected due to their complementary roles in vascular tone regulation, including endothelial relaxation (NO and prostacyclin), vasoconstriction (noradrenaline), and angiogenic signaling (apelin), all of which are known to be altered in PCOS. Given that PCOS is characterized by endothelial dysfunction, chronic low-grade inflammation, and insulin resistance, simultaneous evaluation of these markers allows for a more integrative understanding of the pathophysiological disturbances underlying vascular dysregulation in affected individuals."

This addition aims to enhance the clarity and justification of our biomarker selection.

**Response to Reviewer 4**

***### Reviewer 4 Comment 1: Clinical Implications: The exceptional diagnostic performance of apelin (AUC = 0.983) warrants deeper discussion of its potential clinical utility. How might this biomarker complement existing PCOS diagnostic criteria? Could it help stratify cardiovascular risk?***

We thank the reviewer for this valuable suggestion. To address this comment, we have added a sentence to the Discussion section.

"Given its outstanding diagnostic accuracy (AUC = 0.983), apelin stands out as a particularly promising biomarker. Its high sensitivity and specificity suggest a potential role not only in aiding the diagnosis of PCOS but also in complementing existing criteria such as the Rotterdam criteria. Incorporating apelin into a broader diagnostic panel may enhance diagnostic precision, particularly in patients with atypical or borderline presentations. Moreover, considering its association with vascular dysfunction, apelin could serve as a useful marker for stratifying cardiovascular risk in PCOS patients, paving the way for risk-based clinical management and personalized therapeutic approaches."

***### Reviewer 4 Comment 2: Methodological Details:***

***Specify whether ELISA kits were validated in-house for human serum.***

We thank the reviewer for this valuable suggestion. To address this comment, we have added to “human-specific” phrase relevant section.

***Clarify if blood sampling was timed to specific menstrual phases for cycling controls, as hormonal fluctuations could influence biomarkers.***

We thank the reviewer for this valuable suggestion. To address this comment, we have added a sentence to the relevant section.

"To minimize hormonal variability, blood samples from cycling control participants were collected during the early follicular phase (days 2–5) of the menstrual cycle."

***### Reviewer 4 Comment 3: Results Interpretation:***

***Reconcile the paradoxical findings of elevated NO (vasodilator) and noradrenaline (vasoconstrictor) in PCOS. Is this compensatory? Discuss potential mechanisms.***

We thank the reviewer for this valuable suggestion. To address this comment, we have added a paragraph to the relevant section.

“The concurrent elevation of NO and noradrenaline levels in PCOS may appear paradoxical, given their opposing roles in vascular tone regulation. However, this finding may reflect a compensatory response to ongoing vascular stress or dysfunction. Elevated noradrenaline, indicative of heightened sympathetic activity, could contribute to persistent vasoconstriction and endothelial stress. In response, the endothelium may upregulate NO production via inducible or endothelial NO synthase as a compensatory vasodilatory mechanism aimed at restoring vascular homeostasis. Alternatively, increased NO may be a consequence of oxidative stress and inflammation, common features in PCOS, which stimulate iNOS expression but do not necessarily lead to functional vasodilation. These complex and potentially maladaptive interactions suggest that the vascular system in PCOS operates in a dysregulated state, with both vasodilatory and vasoconstrictive pathways activated simultaneously but ineffectively. This imbalance may contribute to the observed endothelial dysfunction and increased cardiovascular risk in PCOS patients.”

***Address the low AUC for prostacyclin (0.351)—does this suggest limited standalone value, or might it interact with other biomarkers?***

We thank the reviewer for this valuable suggestion. To address this comment, we have added a paragraph to the relevant section.

“While the low AUC indicates that prostacyclin may not be suitable as a standalone diagnostic marker, its well-established role in endothelial function suggests it should not be overlooked. Rather than acting in isolation, prostacyclin may modulate or amplify the effects of other vasoactive molecules. Its reduced levels in PCOS, despite limited predictive power, may reflect a downstream effect of chronic inflammation or hormonal dysregulation. Integrating prostacyclin into multi-marker panels could improve its utility and provide more nuanced insights into PCOS-associated vascular impairment.”

***### Reviewer 4 Comment 4: Writing Refinements:***

***Avoid repetitive phrases (e.g., "vascular tone regulation"); substitute with synonyms like "vascular homeostasis."***

We thank the reviewer for this valuable suggestion. To address this comment, we have added a paragraph to the relevant section.

"While the low AUC indicates that prostacyclin may not be suitable as a standalone diagnostic marker, its well-established role in endothelial function suggests it should not be overlooked. Rather than acting in isolation, prostacyclin may modulate or amplify the effects of other vasoactive molecules. Its reduced levels in PCOS, despite limited predictive power, may reflect a downstream effect of chronic inflammation or hormonal dysregulation. Integrating prostacyclin into multi-marker panels could improve its utility and provide more nuanced insights into PCOS-associated vascular impairment.”

***Define acronyms (e.g., NO, PCOS) only at first use.***

We thank the reviewer for this constructive suggestion. Relevant edits have been made to the entire article.

***### Reviewer 4 Comment 5: Minor Comments:***

***Correct "prostacycline" → "prostacyclin" in Figure 1.***

We thank the reviewer for this valuable suggestion. In Figure 1, the term "prostacycline" has been changed to "prostacyclin".

***Include units in Table 3 column headers rather than the text.***

We appreciate the reviewer’s helpful comment regarding the inclusion of units in the column headers. In Tables 2 and 3, the units have been provided in parentheses immediately following each parameter name to ensure clarity and consistency across all listed variables. We opted for this format to preserve visual alignment and maintain uniformity throughout the tables. However, if the reviewer prefers the units to be placed directly in the column headers, we would be happy to revise the tables accordingly.

***Consider adding a table summarizing biomarker cutoffs, sensitivity/specificity.***

We thank the reviewer for this valuable suggestion. The biomarker cut-off values, along with their corresponding sensitivity and specificity results, have been presented in the text under the “ROC curve analysis” subsection to maintain narrative flow. However, we fully agree that presenting these values in tabular form may enhance clarity and accessibility. If preferred, we would be pleased to reorganize this information into a separate table.
